# Comparative efficacy and safety of metronomic chemotherapy in breast cancer: A protocol for network meta-analysis protocol

**DOI:** 10.1097/MD.0000000000026255

**Published:** 2021-06-11

**Authors:** Ying Xie, Xinjie Chen, Bingxue Li, Xiaoming Wang

**Affiliations:** a Beijing University of Chinese Medicine, Chaoyang District; b Department of Oncology, Beijing Hospital of Traditional Chinese Medicine, Capital Medical University, Dongcheng District, Beijing, China.

**Keywords:** breast cancer, efficacy and safety, metronomic chemotherapy, network meta-analysis

## Abstract

**Background:**

Metronomic chemotherapy (MC) strategy has been used in breast cancer for more than a decade since it was first proposed. The purpose of this study is to systematically evaluate its efficacy and safety for breast cancer patients at various stages, as well as to clarify the most effective medication strategy when applying MC and discover its most sensitive subpopulation in breast cancer patients.

**Method:**

We will systematically retrieve random controlled trials evaluating the efficacy and safety of MC in breast cancer on PubMed, Cochrane Library, Embase, and web of science to perform this network meta-analysis. Markov chain Monte Carlo method based on Bayesian Theory will be used to conduct network meta-analysis and the efficacy and safety will be ranked by combining direct and indirect evidence in mixed treatment comparisons. We will assess the quality of literatures with the Cochrane Risk Bias Assessment Tool and assess the strength of the evidence using the GRADE methodology. Data analysis will be completed with the WinBUGS, R, Stata and RevMan softwares.

**Results and conclusion:**

Through the analysis, we can obtain the ranking of efficacy and safety in different MC strategy, and reveal the specific breast cancer groups that are more sensitive to MC. We access the effectiveness by disease free survival, progress free survival, time to progress, objective response rate, and overall survival, and measure the toxicity by dose-limiting toxicity. The result of our study could provide evidence for clinicians to make a better choice when they consider MC.

**Inplasy registration number:**

INPLASY202140142.

## 1 Introduction

The incidence and mortality of breast cancer, which are respectively 46.3 per 100,000 and 13 per 100,000, rank first in female cancer patients worldwide, according to the data of GLOBOCAN 2018.^[1]^ Treatment of breast cancer has become one of the research hotspots of malignant tumors. In recent years, with the deepening of basic and clinical research on breast cancer, great achievements have been made. However, even though targeted therapy, endocrine therapy and even immunotherapy are explored and developed, chemotherapy is still indispensable in the treatment of breast cancer, so it is very important to optimize the efficacy of chemotherapy as well as lower the toxicity as much as possible.

Metronomic chemotherapy (MC) refers to the strategy of low dose, high frequency and continuous administration of chemotherapy. The concept of “metronomic chemotherapy” was first proposed by Professor Douglas Hanahan in 2000.^[2]^ The first clinical trial tested MC in metastatic breast cancer was published in 2002.^[3]^ From then on, the number of published papers has increased in this field. The results from a national questionnaire among oncologist conducted in Italy indicated a significant interest in MC, with 72% of responders having been administered a regimen of MC at least once.^[4]^ Now MC has become an important strategy in the maintenance treatment of breast cancer.^[5]^ Even more, combination of MC and anti-angiogenic,^[6,7]^ immune therapy^[8,9]^ or applying other targeted therapy in a metronomic way^[10,11]^ enjoy a great popularity in the latest clinical trials. Its application on breast cancer has also attracted more and more attention due to the little drug-related side effects and the high treatment tolerance on patients.^[12]^ However, there is no final conclusion about which strategy provides the best benefit for patients or which subpopulation of breast cancer patient benefit more from MC.

Although there have been several systematic review^[13]^ and meta-analysis^[14]^ related to MC published, it is not clear which strategy is more effective and safe. Here, we provide a protocol to compare the efficacy and safety of various MCs through network meta-analysis to get the optimal treatment regimen.

## 2 Method

### 2.1 Design

Network meta-analysis will use the protocol designed as per the guidelines of preferred reporting items for systematic review and meta-analysis protocol (PRISMA-P).^[15]^

### 2.2 Protocol registration

Our protocol has been registered on INPLASY with identifier INPLASY202140142.

### 2.3 Inclusion criteria

#### 2.3.1 Population

Breast cancer patients of any age, stage or nationality.

#### 2.3.2 Interventions

MC, both monotherapy and combined chemotherapy will be included.

#### 2.3.3 Comparisons

Conventional treatment, guideline recommendations, and expert consensus.

#### 2.3.4 Outcomes

##### 2.3.4.1 Efficacy

Disease free survival is defined as the time from randomization to the first recurrence/metastasis of the tumor or death from any cause; Progress free survival is defined as the time from randomization to the first tumor progression or death; Time to progress is defined as the time from the start of randomization to the first objective progression of the tumor; Objective response rate is defined as the proportion of tumor shrinkage that has reached a certain amount and maintained for a certain time, including complete response and partial response cases; Overall survival is defined as the time from random assignment to death from any cause (the last time of follow-up for lost patients and the end date of follow-up for patients who were still alive at the end of the study)

##### 2.3.4.2 Safety

Proportion of patients experience dose-limiting toxicity is defined as a Grade 3 or 4 non-hematologic safety (excluding alopecia, nausea, and vomiting) or a Grade 4 hematologic safety.^[16]^ We extract the odds ratio value of dose-limiting toxicity between treatment trials.

### 2.4 Search strategy

#### 2.4.1 Bibliographic databases

Electronic searching by titles and abstracts of MC for breast cancers will be performed in PubMed, Cochrane Library, Embase, Web of science and records will be screened with Endnote software.

#### 2.4.2 Search terms

MC, breast cancer, randomized controlled trial. Synonyms for metronome chemotherapy will also be added to the search. Detailed search strategy can be seen in Appendix.

### 2.5 Study selection

Retrieval records will be screened by 2 researchers independently according to the established inclusion criteria. Studies that are not randomized controlled trial and do not contain breast cancer will be excluded by reading title and abstract, and then studies that meet the inclusion criteria and report any of the outcomes of interest were identified by reading the full text. For cases of any disagreement, the two researchers discussed with third opinion until consensus.

### 2.6 Data extraction

Data will be extracted from the eligible studies by two authors independently with same pre-designed data extraction table and the results will be managed with Excel, including the following information:

1. Publication details: year, language, country, authors, journals
2. Baseline factors: Age, marriage and fertility, menopause status, cancer information (including TNM staging information, estrogen receptor, partial response and HER-2 status, pathology grade);
3. Inclusion criteria
4. Outcome indicator and respective odds ratio or hazard ratio value with 95% confidence interval
5. Intervention and comparator details
6. Follow-up time
7. Sample size
8. Number of events in each group

### 2.7 Risk assessment of bias

Two authors will evaluate the quality of the literature separately using the Risk Bias Assessment Tool recommended by Cochrane.^[17]^ For those with different opinions, the third author's opinion will be adopted. The results will be gathered with RevMan software. All studies assessed will be considered in subsequent analysis.

### 2.8 Analysis

#### 2.8.1 Heterogeneity

The results of heterogeneity assessment will be obtained through R software. *I*^2^ ≤ 50% and *P* ≥ .05 indicate that there is no statistical heterogeneity, then a fixed effect model was used to estimate the combined effect size while *I*^2^ > 50% or *P* < .05 indicates the existence of statistical heterogeneity, we should find the cause of heterogeneity and conduct subgroup analysis. If heterogeneity cannot be reduced, a random effects model should be used to estimate the combined effect size.

#### 2.8.2 Consistency

In this study, Loop inconsistency will be analyzed by R software based on the Bucher method.^[18,19]^

#### 2.8.3 Sensitivity

Sensitivity analysis will be conducted in Stata by excluding any of the study.

#### 2.8.4 Model fit test

We will use WinBUGS software to calculate posterior mean of total residual TotresDev, and then compare it with the number of total arms in all trials. If they are similar, fitting is good. According to the value of deviation information criterion, the fixed-effect model and the random effect model can be compared, and the model with smaller deviation information criterion has a better fitting degree.^[20]^

#### 2.8.5 Publication bias

We will use Stata software to conduct Begg rank correlation analysis and Egger linear regression analysis to test whether there is publication bias.

#### 2.8.6 Network meta-analysis

The network geometries are used to show the number of studies and the number of patients included in each intervention.^[21]^ The size of nodes and the thickness of lines in the network diagram respectively represent the number of patients included in the corresponding intervention and the number of studies directly compared with the intervention. R software and WinBUGS software will be used to conduct network meta-analysis based on Markov chain Monte Carlo method of Bayesian theory for a variety of different treatment regimens.^[21,22]^ If the included studies had a good consistency, the efficacy and safety will be ranked by combining the posterior probability of direct comparison evidence and indirect comparison evidence.^[23]^ Estimates of cumulative probability will be sorted based on the probability of each result in a particular ranking (first, second, and so on).

Meta-analysis for the following direct comparison depending on the availability of suitable comparable and meta-analyzable studies.

#### 2.8.7 Comparison

Pair-wise meta-analysis will be proceeded to compare the efficacy and safety of (A) MC and conventional dose chemotherapy with the same regimens; (B) combination of MC and other treatment schedule such as anti-angiogenic therapy, immunotherapy, hormonal therapy and the later themselves. For MC and conventional dose chemotherapy comparison, enumeration data from all control groups were first pooled as the common control, and then network meta-analysis will be conducted. Time-related data will be replaced by dichotomous data at specific time points. For combination of MC and other treatment schedules, trials with common controlled regimens will be included in to network meta-analysis separately. Pairs of regimens comparison will be adjusted according to the actual retrieval results.

### 2.9 Subgroup analysis

We will try to screen out the specific breast cancer population that is more sensitive to MC through subgroup analysis, including their cancer information, TNM, hazard ratio, HER-2, Grade, etc.

### 2.10 Quality of evidence

The quality of evidence will be performed by the grades of recommendation, assessment, development and evaluation (GRADE).^[24]^

## 3 Discussion

MC has its own unique features. In addition to the direct inhibition of tumor cells, it also has anti-angiogenesis and immunomodulation effects,^[25–27]^ because of which researchers tend to combine MC with anti-angiogenic or immune therapy. Conventional chemotherapy regiments prescribe long intervals to allow normal cells to recover and limit side effects, however, this may allow cancer cells to regenerate and acquire resistance.^[28,29]^ MC does not rely on powerful killing effect but inhibits tumor by influencing multiple mechanisms such as apoptosis, senescence, non-apoptotic cell death and immunogenic cell death, anti-angiogenesis and immune regulation, and is less likely to develop drug resistance.^[30,31]^ This may be why MC works better than conventional chemotherapy. Based on low dose, MC induces less toxicity and is Easier to tolerate with, leading to better quality of life.^[12,32,33]^

As attention has been paid on MC regimens for breast cancer, more and more clinical studies have been carried out, providing opportunity for comprehensive evaluation of the efficacy and safety. Until now, relevant system reviews and meta-analyses are crude. With the advantage of indirect comparison of network meta-analysis, we can obtain a larger sample size, compare treatment regimens that cannot be directly compared, try to screen out reliable regimens and specific groups that are more sensitive to MC, so as to provide a basis for clinical practice to select high-quality regimens with better effects, fewer side effects and less cost.

## Author contributions

**Conceptualization:** Ying Xie.

**Data curation:** Ying Xie, Xinjie Chen.

**Funding acquisition:** Xiaomin Wang.

**Methodology:** Ying Xie, Bingxue Li.

**Project administration:** Xiaomin Wang.

**Supervision:** Xiaomin Wang.

**Validation:** Xinjie Chen, Bingxue Li.

**Visualization:** Ying Xie.

**Writing – original draft:** Ying Xie, Xinjie Chen.

**Writing – review & editing:** Xinjie Chen, Bingxue Li, Xiaomin Wang.

## Supplementary Material

Supplemental Digital Content
