# Supplementary material for: Comparative efficacy and safety of metronomic chemotherapy in breast cancer: A protocol for network meta-analysis protocol
Source: Medicine (Baltimore). 2021 Jun 11;100(23):e26255. doi: 10.1097/MD.0000000000026255 (PMC8202618; doi:10.1097/MD.0000000000026255)
Supplement: Supplemental Digital Content [file medi-100-e26255-s001.docx]

Search Stragedy

According to the preliminary literature search, we formulated the retrieval strategy as follow: ((((Capecitabine Maintenance Therapy[Title/Abstract]) OR (((((((((metronomic schedule[Title/Abstract]) OR (antiangiogenic scheduling[Title/Abstract])) OR (metronomic Etoposide[Title/Abstract])) OR (metronomic cisplatin[Title/Abstract])) OR (metronomic gemcitabine[Title/Abstract])) OR (metronomic Vinorelbine[Title/Abstract])) OR (metronomic cyclophosphamide[Title/Abstract])) OR (Antiangiogenic scheduling of chemotherapy[Title/Abstract])) OR (Continuous low-dose therapy[Title/Abstract])) OR (Metronomic chemotherapy[Title/Abstract]) AND (humans[Filter]) AND (humans[Filter])) AND ((((cancer[Title/Abstract]) OR (tumor[Title/Abstract])) OR (carcinoma[Title/Abstract])) OR (neoplasm[Title/Abstract]) AND (humans[Filter]))) AND (((breast[Title/Abstract]) OR (mammary[Title/Abstract])) AND (humans[Filter]))) AND (((randomized controlled trial[Publication Type]) OR (randomized[Title/Abstract])) OR (placebo[Title/Abstract]) AND (humans[Filter])).

This search strategy

This retrieval strategy was determined based on the PICOS principle, of which the comparison intervention will not be limited and the outcomes for selected studies will be manually confirmed with a deliberate secondary literature search.
